# Association of thyroid nodules with adiposity: a community-based cross-sectional study in China

**DOI:** 10.1186/s12902-018-0232-8

**Published:** 2018-01-27

**Authors:** Bin Song, Zhihua Zuo, Juan Tan, Jianjin Guo, Weiping Teng, Yibing Lu, Chao Liu

**Affiliations:** 1 Department of Endocrinology, The Second Affiliated Hospital of Nanjing Medical University, 125 Jiangjiayuan Road, Nanjing, 211166 China; 2 Department of Endocrinology, Clinical Medical College, Yangzhou University, 98 Nantong West Road, Yangzhou, 225001 China; 3 Department of Gerontology, Huai’an First People’s Hospital, Nanjing Medical University, 6 Beijing West Road, Huai’an, 223300 China; 4 Department of Endocrinology, Second Hospital of Shanxi Medical University, 382 Wuyi Road, Taiyuan, 030001 China; 5 Department of Endocrinology and Metabolism, The First Hospital of China Medical University, 155 Nanjing Road, Shenyang, 110001 China; 6 Endocrine and Diabetes Center, Affiliated Hospital of Integrated Traditional Chinese and Western Medicine, Nanjing University of Chinese Medicine, 8 Huadian East Road, Nanjing, 210028 China

**Keywords:** Thyroid nodules, Body mass index, Overweight, Waist circumference, Central obesity

## Abstract

**Background:**

The association between thyroid nodules and adiposity remains controversial. We performed a cross-sectional, community-based study to examine whether thyroid nodules are associated with overweight and obesity, as defined with body mass index (BMI) and waist circumference.

**Methods:**

The study included 1482 subjects (≥20 years of age; residing in Nanjing, China) receiving questionnaire interview, anthropometric measurements, laboratory tests and thyroid ultrasonography in 2009–2010. Overweight and obesity were defined as BMI ≥24 and ≥28 kg/m^2^, respectively. Central obesity was defined as waist circumference at ≥90 cm in men and ≥80 cm in women. A sensitivity analysis was conducted using the American Diabetes Association (ADA) criteria for overweight and obesity (BMI ≥ 23 and ≥25 kg/m^2^).

**Results:**

Thyroid nodules were identified in 12.6% of the subjects. A greater proportion of the subjects with thyroid nodules had a BMI at ≥24 kg/m^2^ (51.9% vs. 40.5% in those without thyroid nodules, *P =* 0.003) and central obesity (43.3% vs. 24.2%, *P* < 0.001). After adjustment for other confounders, central obesity was still associated with significantly elevated risk of thyroid nodules (OR 1.62, 95%CI 1.14–2.28), whereas obesity/overweight based on BMI was not in both the main analysis and sensitivity analysis with the alternative criteria. In the subgroup analysis, BMI ≥24 kg/m^2^ (OR 1.61, 95%CI 1.01–2.54), as well as BMI ≥25 kg/m^2^ (OR 1.95, 95%CI 1.14–3.34), was significantly associated with higher risk of thyroid nodules among women. Using the ADA criteria, overweight and obesity were associated with thyroid nodules (OR 5.59, 95%CI 1.39–22.51 and 5.15, 95%CI 1.30–20.37) in thyroid-stimulating hormone (TSH) > 4.2 mIU/L subgroup. Central obesity correlated with higher risk of thyroid nodules regardless of age (< 50 years: OR 1.87, 95%CI 1.05–3.32: ≥50 years: OR 1.54, 95%CI 1.00–2.37) and in the following subgroups: men (OR 1.91, 95%CI 1.14–3.20), TSH > 4.2 mIU/L (OR 3.05, 95%CI 1.01–9.22), and urine iodine ≥200 *µ*g/L (OR 1.79, 95%CI 1.14–2.81).

**Conclusion:**

Waist circumference is superior to BMI for assessing risk of thyroid nodules in Chinese subjects.

**Electronic supplementary material:**

The online version of this article (10.1186/s12902-018-0232-8) contains supplementary material, which is available to authorized users.

## Background

Thyroid nodules are one of the most common thyroid diseases and their incidence has been rising in recent decades worldwide. Although most thyroid nodules are benign, detecting them early is important because there is always risk that the nodules may be cancerous [1]. Several factors have been associated with the formation of thyroid nodules, including gender [2–4], age [2–9], thyroid-stimulating hormone (TSH) [9], and iodine intake [10, 11]. Numerous studies have also associated thyroid nodules with adiposity [3–5, 8, 9, 12–14], which is traditionally evaluated based on body mass index (BMI). While some studies have supported a positive correlation between BMI and risk of thyroid nodules [3, 5, 13], particularly in women, other studies have failed to detect this association [2, 6, 7], including our own work with children [15]. In addition, two case–control studies from different countries found that morbid obesity in women (BMI ≥40 kg/m^2^) was associated with lower prevalence of thyroid nodules [12, 14]. These findings call into question whether BMI is useful as a predictor of thyroid nodule risk.

Another measure of adiposity is waist circumference. While BMI cannot distinguish between general adiposity and central (or abdominal) obesity, waist circumference reflects specifically central obesity. In several types of chronic diseases, including cardiovascular disease [16], renal disease [17] and metabolic syndrome [18], central obesity correlates more strongly with adverse health outcomes than higher BMI. Several studies have associated metabolic syndrome with increased prevalence of thyroid nodules [2, 4, 8, 9, 19], and one of these studies found waist circumference to correlate positively with thyroid nodules in men, although not in women [9].

Whether central obesity is associated with thyroid nodules is unclear. Studies from our group [4] and others [8] involving individuals showing moderate iodine intake have linked central obesity to higher prevalence of thyroid nodules, whereas another study of individuals with mild to moderate iodine deficiency failed to find this association after adjusting for insulin resistance [19]. These discrepancies may reflect differences in study design, sample size, ethnicity, gender and iodine levels.

Therefore we undertook the present study to clarify whether central obesity is significantly associated with the presence of thyroid nodules and may therefore serve as a useful indicator of thyroid nodule risk. In addition, we compared the association of thyroid nodule risk with higher waist circumference or elevated BMI in order to determine whether one of these adiposity indicators is superior to the other. Our study population came from our previous study designed to investigate thyroid diseases and iodine nutrition in a Chinese community-based population. Median urinary iodine concentration in this population was 239 *µ*g/L, indicating more than adequate iodine intake [11].

## Methods

### Subjects

Participants in the present study were taken from the population analyzed in our multicenter, cross-sectional epidemiological investigation of thyroid diseases in 10 Chinese cities [11]. From among this population originally recruited in 2009–2010, we selected a community in Nanjing where most inhabitants had lived for more than 5 years. To avoid recruitment bias, we screened the residents based on their household registrations and stratified sampling by age to mirror the average age composition of Chinese urban populations, based on 2008 data from the Chinese National Bureau of Statistics. This led us to enroll 1572 individuals older than 20 years old (men: women, 1:1.2), who showed the following age distribution: 20–29 years, 17.5%; 30–39 years, 23.2%; 40–49 years, 23.3%; 50–59 years, 17.9%; 60–69 years, 10.1%; ≥70 years, 7.9%.

Participants were excluded if they were pregnant women or women who had given birth during the preceding year; if they had adrenocortical insufficiency, renal insufficiency or other serious systemic disease; or if they were receiving any treatment that might affect thyroid function and iodine excretion, such as glucocorticoids, antiepileptic drugs, amiodarone or iodine-containing contrast agents. Details of the study design, eligibility, recruitment, survey procedures and participant characteristics were reported elsewhere [11, 20]. This study was conducted in accordance with the Declaration of Helsinki and approved by the medical ethics committee of China Medical University (serial number: IRB [2008]34) [11]. Written informed consent was obtained from all participants before any sample or data collection.

### Data collection

As previously described [11], a structured questionnaire was administered by trained staff during a face-to-face interview in order to collect data on demographic characteristics, smoking status, dietary habits, type of salt used, and personal or family history of thyroid disease. Physical examination included weight (measured to nearest 0.5 kg), height (nearest 0.1 cm), waist circumference (nearest 0.1 cm) and blood pressure (nearest 1 mmHg). Height and weight were evaluated using a calibrated balance beam scale when subjects wore light clothes and were barefoot. Waist circumference was measured midway between the lowest rib and iliac crest using a tape measure while participants were standing and breathing normally. Systolic and diastolic blood pressure was measured using a standard manual mercury sphygmomanometer while the subject was seated. Pressure was measured twice at an interval of 30 s, and the readings were averaged. Overnight fasting blood and urine samples from all subjects were assayed for serum levels of TSH, thyroid peroxidase antibodies (TPOAb), thyroglobulin antibodies (TgAb) and urine iodine concentration (UIC). Normal reference ranges were 0.27–4.2 mIU/L for TSH, 0–34 IU/L for TPOAb and 0–115 IU/L for TgAb [11]. Thyroid ultrasonography was performed by professional physicians who had received centralized training using a portable instrument (LOGIQ a50, 7.5 MHz; GE Healthcare).

### Definition of variables

A thyroid nodule was defined as a discrete lesion that was distinct from the surrounding thyroid parenchyma and that had a solid portion, regardless of whether a cystic portion was present [21]. Central obesity was defined as waist circumference ≥ 90 cm for men or ≥80 cm for women, based on the criteria recommended for Chinese adults by the International Diabetes Federation [22]. BMI was calculated as weight (kg) divided by height (m) squared, and definition of overweight and obesity was BMI ≥ 24 and ≥28 kg/m^2^, respectively, according to the 2016 China consensus statement on management of overweight/obesity [23]. Additionally, a sensitivity analysis was conducted using the American Diabetes Association definition for overweight (≥23 kg/m^2^) and obesity (≥25 kg/m^2^) for Asians [24]. Elevated TSH was defined as > 4.2 mIU/L, and the presence of antithyroid antibodies was defined as TPOAb > 34 IU/L or TgAb > 115 IU/L. UIC was classified as low if < 200 *µ*g/L and high if ≥200 *µ*g/L based on World Health Organization guidelines.

### Statistical analysis

Continuous data are reported as mean ± standard deviation (SD) or median (interquartile range), while categorical data are reported as count and percentage. Differences in means, medians or proportions between individuals with or without thyroid nodules were assessed for significance using the Mann–Whitney U and chi-squared tests. Logistic regression was used to calculate odds ratios (ORs) and 95% confidence intervals (95%CIs) to assess the association of thyroid nodules with waist circumference or BMI. Adjusted logistic regression models took into account age, gender, education, profession, smoking status, systolic and diastolic blood pressure, TSH and UIC.

Subgroup analyses were performed by treating BMI and waist circumference as categorical variables and stratifying subjects based on four potential risk factors for thyroid nodules: gender, age (< 50 or ≥50 years), TSH (≤4.2 or > 4.2 mIU/L), and UIC (< 200 or ≥200 *µ*g/L). Heterogeneity in the influence of BMI or waist circumference on thyroid nodules between participants with or without these factors was evaluated by adding an interaction term to the relevant adjusted model. All analyses were performed using EmpowerStats (http://www.empowerstats.com; X&Y Solutions, Boston, MA). A two-sided significance level of 0.05 was used to evaluate statistical significance.

## Results

### Baseline characteristics

Of the 1572 individuals recruited, 40 were excluded from the study because they did not complete the survey, resulting in a response rate of 97.4%. We excluded another 50 participants because of previous thyroid disease. In the end, 1482 subjects were included in the study (mean age, 44.5 ± 15.4 years), of whom 682 (46.0%) were males and 187 (12.6%) had thyroid nodules (Table 1). Mean BMI was 23.52 ± 3.33 kg/m^2^ and mean waist circumference was 79.68 ± 9.56 cm; 41.9% of subjects had BMI ≥ 24 kg/m^2^, and 26.7% had central obesity based on waist circumference. Individuals with thyroid nodules were significantly older than those without nodules (54.6 vs. 43.0 years, *P* < 0.001) and had significantly higher BMI (24.10 vs. 23.44 kg/m^2^, *P* = 0.011) and waist circumference (82.38 vs. 79.29 cm, *P* = 0.030), as well as significantly lower TSH (2.17 vs. 2.52 mIU/L, *P* < 0.001). The proportion of individuals with BMI ≥ 24 kg/m^2^ was significantly higher among individuals with nodules (51.9% vs. 40.5%, *P =* 0.003), as was the proportion of individuals with central obesity (43.3% vs. 24.2%, *P* < 0.001). Individuals with or without thyroid nodules were similar in terms of gender composition, UIC, smoking status, family history of thyroid disease, iodized salt intake, seafood intake, and TPOAb or TgAb positivity.

**Table 1:** Baseline characteristics of a community-based Chinese population with or without thyroid nodules

Characteristic	Total (*n* = 1482)	No nodules (*n* = 1295)	Nodules (*n* = 187)	*P*
Male	682 (46.0)	605 (46.7)	77 (41.2)	0.155
Age (years)	44.5 ± 15.4	43.0 ± 14.8	54.6 ± 15.3	< 0.001
BMI (kg/m^2^)	23.52 ± 3.33	23.44 ± 3.35	24.10 ± 3.18	0.011
WC (cm)	79.68 ± 9.56	79.29 ± 9.53	82.38 ± 9.36	< 0.001
SBP (mmHg)	123.22 ± 16.83	122.38 ± 16.43	129.06 ± 18.38	< 0.001
DBP (mmHg)	78.42 ± 10.74	78.11 ± 10.70	80.55 ± 10.76	0.004
UIC (μg/L)	240.6 (163.6–336.5)	243.0 (165.2–338.1)	222.0 (151.6–328.6)	0.193
TSH (mIU/L)	2.48 (1.67–3.46)	2.52 (1.71–3.45)	2.17 (1.44–3.59)	0.030
Education				< 0.001
Primary school or illiterate	132 (8.9)	106 (8.2)	26 (13.9)	
Junior high school	399 (26.9)	334 (25.8)	65 (34.8)	
Senior high school	697 (47.0)	627 (48.4)	70 (37.4)	
Undergraduate or above	254 (17.1)	228 (17.6)	26 (13.9)	
Profession				< 0.001
Employed	1045 (70.5)	947 (73.1)	98 (52.4)	
Unemployed	354 (23.9)	271 (20.9)	83 (44.4)	
Not reported	83 (5.6)	77 (5.9)	6 (3.2)	
Smoking status				0.187
Never	1084 (73.1)	937 (72.4)	147 (78.6)	
Currently	386 (26.0)	347 (26.8)	39 (20.9)	
Formerly	12 (0.8)	11 (0.8)	1 (0.5)	
Family history of thyroid disease	43 (2.9)	35 (2.7)	8 (4.3)	0.241
Iodized salt	1450 (97.8)	1266 (97.8)	184 (98.4)	0.789
Seafood intake				0.851
Never	83 (5.6)	73 (5.6)	10 (5.3)	
Occasionally	1166 (78.7)	1021 (78.8)	145 (77.5)	
Frequently	233 (15.7)	201 (15.5)	32 (17.1)	
TPOAb positive	139 (9.4)	122 (9.4)	17 (9.1)	0.885
TgAb positive	163 (11.9)	141 (10.9)	22 (11.8)	0.720
BMI ≥24 kg/m^2^	621 (41.9)	524 (40.5)	97 (51.9)	0.003
Central obesity	395 (26.7)	314 (24.2)	81 (43.3)	< 0.001

Continuous data are shown as the mean ± standard deviation or median (interquartile), and categorical data as n (%)

### Associations of thyroid nodules with waist circumference and BMI

Associations of BMI and waist circumference with thyroid nodules across the entire study population are shown in Table 2. BMI considered as a continuous variable (per 1 kg/m^2^ increase) was associated with increased risk of thyroid nodules in the unadjusted model (OR 1.06, 95%CI 1.01–1.11), but this association was no longer significant after adjusting for age, gender, education, profession, smoking status, systolic and diastolic pressure, TSH and UIC (OR 1.02, 95%CI 0.97–1.07). BMI as a categorical variable was also associated with elevated risk of thyroid nodules: the OR for individuals with BMI ≥24 kg/m^2^relative to those with BMI < 24 kg/m^2^ was 1.59 (95%CI 1.17–2.16) in the unadjusted model. Again, this association was not significant in the adjusted model (OR 1.33, 95%CI 0.95–1.86). Additionally, using the ADA definition for overweight (≥23 kg/m^2^) and obesity (≥25 kg/m^2^) for Asians, results are consistent and shown in Additional file 1: Table S1. In the unadjusted model, the ORs for individuals with BMI ≥23, < 25 kg/m^2^ and BMI ≥25 kg/m^2^ relative to those with BMI < 23 kg/m^2^ were 1.62 (95%CI 1.10–2.40) and 1.70 (95%CI 1.19–2.44), respectively. Those associations were not significant in the adjusted model (OR 1.47, 95%CI 0.97–2.23 and OR 1.43, 95%CI 0.96–2.13, respectively).

**Table 2:** Analysis of associations of thyroid nodules with BMI and waist circumference

Predictor	Unadjusted OR, 95%CI	*P*	^a^Adjusted OR, 95%CI	*P*
BMI (per kg/m^2^)	1.06 (1.01, 1.11)	0.011	1.02 (0.97, 1.07)	0.459
BMI				
< 24 kg/m^2^	Ref		Ref	
≥ 24 kg/m^2^	1.59 (1.17, 2.16)	0.003	1.33 (0.95, 1.86)	0.100
Waist circumference (per cm)	1.03 (1.02, 1.05)	< 0.001	1.02 (1.00, 1.04)	0.034
Central obesity				
No	Ref		Ref	
Yes	2.39 (1.74, 3.27)	< 0.001	1.62 (1.14, 2.28)	0.007

^a^The adjusted OR controls for age, gender, education, profession, smoking status, systolic and diastolic blood pressure, TSH, and UIC

Waist circumference considered as a continuous variable (per 1 cm increase) was significantly associated with increased risk of thyroid nodules in an unadjusted model (OR 1.03, 95%CI 1.02–1.05) as well as in the adjusted model (OR 1.02, 95%CI 1.00–1.04). Central obesity, defined according to a gender-specific waist circumference cut-off, was significantly associated with higher risk of thyroid nodules in the unadjusted model (OR 2.39, 95%CI 1.74–3.27) and adjusted model (OR 1.62, 95%CI 1.14–2.28).

### Subgroup analyses based on gender, age, TSH and UIC

Possible relationships of thyroid nodules with BMI or waist circumference, both considered as categorical variables, were explored in different subgroups of study participants (Table 3). BMI ≥ 24 kg/m^2^ was significantly associated with higher thyroid nodule risk only in women (OR 1.61, 95%CI 1.01–2.54), while central obesity significantly correlated with increased risk of thyroid nodules in men (OR 1.91, 95%CI 1.14–3.20), individuals younger than 50 years (OR 1.87, 95%CI 1.05–3.32), individuals at least 50 years (OR 1.54, 95%CI 1.00–2.37), individuals with TSH > 4.2 mIU/L (OR 3.05, 95%CI 1.01–9.22) and UIC ≥200 *µ*g/L (OR 1.79, 95%CI, 1.14–2.81). Neither BMI ≥ 24 kg/m^2^ nor central obesity interacted significantly with any of the four potential thyroid nodule risk factors of gender, age, TSH, or UIC (all *P* for interaction > 0.05).

**Table 3:** Associations of thyroid nodules with BMI and waist circumference in subgroups of subjects stratified by gender, age, TSH or UIC

Predictor		BMI ≥24 kg/m^2^			Central obesity		
	N	Adjusted OR, 95%CI	*P*	*P* interaction	Adjusted OR, 95%CI	*P*	*P* interaction
Gender^a^							
Male	682	1.14 (0.68, 1.90)	0.626	0.372	1.91 (1.14, 3.20)	0.013	0.272
Female	800	1.61 (1.01, 2.54)	0.044		1.44 (0.89, 2.35)	0.142	
Age^b^							
< 50 years	958	1.61 (0.94, 2.74)	0.081	0.185	1.87 (1.05, 3.32)	0.033	0.632
≥ 50 years	524	1.14 (0.74, 1.75)	0.559		1.54 (1.00, 2.37)	0.048	
TSH^c^							
≤ 4.2 mIU/L	1259	1.17 (0.81, 1.68)	0.408	0.161	1.41 (0.97, 2.05)	0.069	0.194
> 4.2 mIU/L	223	2.52 (0.91, 6.99)	0.076		3.05 (1.01, 9.22)	0.048	
UIC^d^							
< 200 *µ*g/L	543	1.08 (0.63, 1.85)	0.785	0.375	1.30 (0.75, 2.24)	0.351	0.373
≥ 200 *µ*g/L	939	1.48 (0.95, 2.29)	0.082		1.79 (1.14, 2.81)	0.012	

^a^Adjusted for age, education, profession, smoking status, systolic and diastolic blood pressure, TSH, and UIC

^b^adjusted for gender, education, profession, smoking status, systolic and diastolic blood pressure, TSH, and UIC

^c^adjusted for age, gender, education, profession, smoking status, systolic and diastolic blood pressure, and UIC

^d^adjusted for age, gender, education, profession, smoking status, systolic and diastolic blood pressure, and TSH

Additionally, in the sensitivity analysis according to ADA definition for Asians (Additional file 2: Table S2), compared with BMI < 23 kg/m^2^, BMI ≥ 25 kg/m^2^ was significantly associated with higher thyroid nodule risk among women (OR 1.95, 95%CI 1.14–3.34) and individuals with TSH > 4.2 mIU/L (OR 5.15, 95%CI 1.30–20.37). Similarly, individuals with BMI ≥23, < 25 kg/m^2^ relative to those with BMI < 23 kg/m^2^ was 5.59 (95%CI 1.39–22.51) in TSH > 4.2 mIU/L subgroup. Interestingly, BMI based on 23 and 25 kg/m^2^ interacted significantly with TSH (*P* for interaction = 0.044).

## Discussion

In this cross-sectional study of a community-based population in China showing more than adequate iodine intake, we were unable to confirm a significant association between high BMI and risk of thyroid nodules, except for the subgroup of women. In contrast, we found an independent, positive correlation between waist circumference – treated as a categorical or continuous variable – and risk of thyroid nodules. Participants with central obesity were at 1.62-fold higher risk of thyroid nodules than those with normal waist circumference, and this relationship was also observed in nearly all subgroup analyses. Our findings suggest that in Chinese individuals with more than adequate iodine intake, higher waist circumference is more strongly associated than higher BMI with elevated risk of thyroid nodules. This may mean that adipose tissue in the waist area may influence risk of thyroid nodules differently from adipose tissue elsewhere in the body.

Previous studies in different populations have reached conflicting conclusions about the association between BMI and risk of thyroid nodules. Two community-based studies in China reported that overweight and general obesity (as measured using BMI) were associated with higher risk of thyroid nodules only in women [5, 13], and we found the same result in our population when we treated BMI as a categorical variable. However, another Chinese study did not detect this association, perhaps because of insufficient sample size [2]. A previous community-based study in China found that BMI defined as a continuous variable correlated positively with risk of thyroid nodules [3], but two other studies involving healthy individuals undergoing physical exams failed to detect this association [6, 7], similar to our negative result in the present study. To make things more complicated, two studies outside Asia reported a negative relationship between BMI and risk of thyroid nodules [12, 14]. In light of the literature, we speculate that BMI may not correlate linearly with thyroid nodule risk, and so it may be unsuitable for assessing the influence of adiposity on the presence of thyroid nodules. Whether this is true only for Chinese populations or more broadly requires further study.

The poor performance of BMI as an indicator of thyroid nodules may relate to the fact that it is a quite nonspecific measure of adiposity, aggregating measures of muscle mass, peripheral and abdominal adipose tissue, and bone mass [17]. Waist circumference, in contrast, specifically reflects abdominal adipose distribution, which mainly consists of subcutaneous and visceral adipose tissue [25, 26]. This specificity may help to explain why waist circumference appears to be a better indicator of thyroid nodule risk. Central obesity has already been linked to greater likelihood of adverse metabolic health conditions [18, 27], including hyperglycemia, hypertension and dyslipidemia, which reflect a cluster of components in metabolic syndrome. Only approximately 20% of obese individuals (based on BMI) have metabolic disorders because of their smaller proportion of visceral adipose tissue [25]. In other words, larger waist circumference appears to be a stronger risk factor than BMI for metabolic syndrome [18]. Our finding of a strong correlation between waist circumference and thyroid nodule risk may therefore reflect the well-established correlation between metabolic syndrome and thyroid nodule risk [2, 4, 8, 9, 19]. Indeed, several of those previous studies have reported correlations between central obesity and thyroid nodule risk [4, 8, 9]. Central obesity lies at the core of metabolic syndrome [22], so it may not be surprising that our results show central obesity to be more closely associated with thyroid nodules than overweight and general obesity.

In our association analyses, we took into account age, gender, TSH and UIC as potential confounders. Thyroid nodules are known to be more prevalent in women [2–4] and older individuals [2–9], while waist circumference tends to larger in males and in the elderly. Thyroid nodule formation has been associated with TSH [9] and UIC [10, 11], and TSH has been associated with waist circumference [28]. Our observation that the increased risk of thyroid nodules was markedly attenuated after adjustment for these potential confounders suggests that these covariates also contribute to overall risk. Subgroup analyses showed that central obesity was significantly and independently associated with higher risk of thyroid nodules in nearly all subgroups, while BMI ≥24 kg/m^2^ significantly correlated with increased risk of thyroid nodules only in women. This provides further evidence that, overall, central obesity is more strongly associated with risk of thyroid nodules than overweight and general obesity. The present study shows no evidence that gender, age, TSH, or UIC affects the observed relationship between risk of nodules and either BMI ≥ 24 kg/m^2^ or central obesity. Certainly, according to American Diabetes Association definition for overweight (≥23 kg/m^2^) and obesity (≥25 kg/m^2^) for Asians [24], higher BMI was also significantly associated with higher thyroid nodule risk among individuals with TSH > 4.2 mIU/L, and TSH played an interactive role in the association between BMI and TNs, which might deserve further researches.

We found central obesity to be significantly related to higher thyroid nodule risk in men but not in women. While further work is needed to confirm that this is not merely a sample size effect, we suggest that it may reflect sex differences in the proportion of abdominal fat components. Women tend to have larger stores of subcutaneous fat than visceral fat, while men tend to have more visceral fat than subcutaneous fat for any given waist circumference [25, 26]. This implies that increasing waist circumference represents, in men, primarily accumulation of visceral fat. Obese individuals with greater proportion of subcutaneous fat than visceral fat are at lower risk of metabolic syndrome than those with more visceral than subcutaneous fat [25]. These findings, when taken together with our present results, suggest that waist circumference-associated visceral fat may play a key role in the development of thyroid nodules.

These considerations may point to a key role of insulin resistance in formation of thyroid nodules. Visceral fat is the strongest predictor of insulin resistance [22, 25], which is a central contributor to metabolic syndrome [22]. One study in Italy found that while waist circumference was significantly associated with the presence of thyroid nodules, this association was no longer significant after adjusting for insulin resistance [19]. These results suggest that insulin resistance may be associated with thyroid nodule formation more strongly than even waist circumference. Indeed, our previous study of a large population suggested that insulin resistance is associated with the distribution, construction, and density of blood vessels in thyroid nodules [29]. Differences in such vascularization may help determine nodule growth and progression. It is possible that insulin resistance may cause changes in proliferative pathways activated directly by insulin or insulin-like growth factor-1 (IGF-1), which helps regulate thyroid gene expression and may be important in thyrocyte proliferation and differentiation [4, 30, 31]. Previous studies and the present work argue for focusing future research on the potential role of waist circumference-associated insulin resistance in the formation of thyroid nodules. The available evidence further suggests that effective diagnosis and treatment of insulin resistance may help prevent such nodules.

The present study extends previous work on associations of thyroid nodules with BMI or waist circumference to the case of a population with more than adequate iodine intake (UIC = 239 *µ*g/L). In addition, our study was able to show that waist circumference was associated with thyroid nodules independently of TSH and UIC. These two thyroid nodule risk factors are usually ignored as potential confounders in the literature. Finally, our study systematically compared two adiposity measures, whereas most previous studies have focused on one or the other.

Nevertheless, the results of the present work should be interpreted with caution in light of several limitations. First, the cross-sectional study design does not allow us to establish causal links between obesity measures and thyroid nodules. Second, although the multivariate model adjusted for as many confounders as possible, we did not control for other components of metabolic syndrome, including hyperglycemia, hypertension and dyslipidemia. This reflects the fact that the original purpose of our study was to investigate relationships between iodine nutrition and thyroid diseases, so we did not collect information about history of hypertension, diabetes or dyslipidemia. Third, we did not examine whether the relationship of thyroid nodule risk to BMI or waist circumference depends on the specific nodule subtype.

## Conclusions

In this community-based Chinese population showing more than adequate iodine intake, waist circumference was consistently and more strongly associated than BMI with higher risk of thyroid nodules. These results suggest that waist circumference may be the better indicator of thyroid nodule risk. Maintaining normal waist circumference may help prevent thyroid nodules, particularly in men.

## Additional files

Additional file 1: Table S1. Analysis of associations of thyroid nodules with different BMI cut-offs. (DOCX 19 kb)

Additional file 2: Table S2. Associations of thyroid nodules with different BMI cut-offs in subgroups of subjects stratified by gender, age, TSH or UIC. (DOCX 16 kb)

## Abbreviations

ADA: American Diabetes Association
BMI: body mass index
CI: Confidence interval
DBP: Diastolic blood pressure
IGF-1: Insulin-like growth factor-1
OR: Odds ratio
SBP: Systolic blood pressure
TgAb: Thyroglobulin antibodies
TPOAb: Thyroid peroxidase antibodies
TSH: Thyroid-stimulating hormone
UIC: Urine iodine concentration
WC: Waist circumference

## References

[1] Burman KD, Wartofsky L (2015). CLINICAL PRACTICE. Thyroid nodules. N Engl J Med.

[2] Guo H, Sun M, He W, Chen H, Li W, Tang J, Tang W, Lu J, Bi Y, Ning G (2014). The prevalence of thyroid nodules and its relationship with metabolic parameters in a Chinese community-based population aged over 40 years. Endocrine.

[3] Jiang H, Tian Y, Yan W, Kong Y, Wang H, Wang A, Dou J, Liang P, Mu Y (2016). The prevalence of thyroid nodules and an analysis of related lifestyle factors in Beijing communities. Int J Environ Res Public Health.

[4] Feng S, Zhang Z, Xu S, Mao X, Feng Y, Zhu Y, Liu C (2017). The prevalence of thyroid nodules and their association with metabolic syndrome risk factors in a moderate iodine intake area. Metab Syndr Relat Disord.

[5] Xu W, Chen Z, Li N, Liu H, Huo L, Huang Y, Jin X, Deng J, Zhu S, Zhang S (2015). Relationship of anthropometric measurements to thyroid nodules in a Chinese population. BMJ Open.

[6] Kim JY, Jung EJ, Park ST, Jeong SH, Jeong CY, Ju YT, Lee YJ, Hong SC, Choi SK, Ha WS (2012). Body size and thyroid nodules in healthy Korean population. J Korean Surg Soc.

[7] Sharen G, Zhang B, Zhao R, Sun J, Gai X, Lou H (2014). Retrospective Epidemiological study of thyroid nodules by ultrasound in asymptomatic subjects. Chin Med J.

[8] Yin J, Wang C, Shao Q, Qu D, Song Z, Shan P, Zhang T, Xu J, Liang Q, Zhang S (2014). Relationship between the prevalence of thyroid nodules and metabolic syndrome in the iodine-adequate area of Hangzhou, China: a cross-sectional and cohort study. Int J Endocrinol.

[9] Shin J, Kim MH, Yoon KH, Kang MI, Cha BY, Lim DJ (2016). Relationship between metabolic syndrome and thyroid nodules in healthy Koreans. Korean J Intern Med.

[10] Chen Z, Xu W, Huang Y, Jin X, Deng J, Zhu S, Liu H, Zhang S, Yu Y (2013). Associations of noniodized salt and thyroid nodule among the Chinese population: a large cross-sectional study. Am J Clin Nutr.

[11] Shan Z, Chen L, Lian X, Liu C, Shi B, Shi L, Tong N, Wang S, Weng J, Zhao J (2016). Iodine status and prevalence of thyroid disorders after introduction of mandatory universal salt iodization for 16 years in China: a cross-sectional study in 10 cities. Thyroid.

[12] Cappelli C, Pirola I, Mittempergher F, De Martino E, Casella C, Agosti B, Nascimbeni R, Formenti A, Rosei EA, Castellano M (2012). Morbid obesity in women is associated to a lower prevalence of thyroid nodules. Obes Surg.

[13] Zheng L, Yan W, Kong Y, Liang P, Mu Y (2015). An epidemiological study of risk factors of thyroid nodule and goiter in Chinese women. Int J Environ Res Public Health.

[14] Sousa PA, Vaisman M, Carneiro JR, Guimarães L, Freitas H, Pinheiro MF, Liechocki S, Monteiro CM, Teixeira PF (2013). Prevalence of goiter and thyroid nodular disease in patients with class III obesity. Arq Bras Endocrinol Metabol.

[15] Chen H, Zhang H, Tang W, Xi Q, Liu X, Duan Y, Liu C (2013). Thyroid function and morphology in overweight and obese children and adolescents in a Chinese population. J Pediatr Endocrinol Metab.

[16] Lo K, Wong M, Khalechelvam P, Tam W (2016). Waist-to-height ratio, body mass index and waist circumference for screening paediatric cardio-metabolic risk factors: a meta-analysis. Obes Rev.

[17] Kramer H, Gutiérrez OM, Judd SE, Muntner P, Warnock DG, Tanner RM, Panwar B, Shoham DA, McClellan W (2016). Waist circumference, body mass index, and ESRD in the REGARDS (reasons for geographic and racial differences in stroke) study. Am J Kidney Dis.

[18] Zhang P, Wang R, Gao C, Jiang L, Lv X, Song Y, Li B (2016). Prevalence of central obesity among adults with normal BMI and its association with metabolic diseases in Northeast China. PLoS One.

[19] Ayturk S, Gursoy A, Kut A, Anil C, Nar A, Tutuncu NB (2009). Metabolic syndrome and its components are associated with increased thyroid volume and nodule prevalence in a mild-to-moderate iodine-deficient area. Eur J Endocrinol.

[20] Yan YR, Liu Y, Huang H, Lv QG, Gao XL, Jiang J, Tong NW (2015). Iodine nutrition and thyroid diseases in Chengdu, China: an epidemiological study. QJM.

[21] Cooper DS, Doherty GM, Haugen BR, Hauger BR, Kloos RT, Lee SL, Mandel SJ, Mazzaferri EL, McIver B, Pacini F (2009). Revised American Thyroid Association management guidelines for patients with thyroid nodules and differentiated thyroid cancer. Thyroid.

[22] Alberti KG, Zimmet P, Shaw J (2005). The metabolic syndrome--a new worldwide definition. Lancet.

[23] Chen W (2016). Consensus statement of the Chinese medical and nutritional experts on management for overweight/obesity in China (2016). Chin J Diabetes Mellitus.

[24] American Diabetes Association (2017). Standards of medical Care in Diabetes-2017 abridged for primary care providers. Clin Diabetes.

[25] Power ML, Schulkin J (2008). Sex differences in fat storage, fat metabolism, and the health risks from obesity: possible evolutionary origins. Br J Nutr.

[26] Lemieux S, Prud'homme D, Bouchard C, Tremblay A, Després JP (1993). Sex differences in the relation of visceral adipose tissue accumulation to total body fatness. Am J Clin Nutr.

[27] Janssen I, Katzmarzyk PT, Ross R (2004). Waist circumference and not body mass index explains obesity-related health risk. Am J Clin Nutr.

[28] Mamtani M, Kulkarni H, Dyer TD, Almasy L, Mahaney MC, Duggirala R, Comuzzie AG, Samollow PB, Blangero J, Curran JE (2014). Increased waist circumference is independently associated with hypothyroidism in Mexican Americans: replicative evidence from two large, population-based studies. BMC Endocr Disord.

[29] Wang K, Yang Y, Wu Y, Chen J, Zhang D, Mao X, Wu X, Long X, Liu C (2015). The association between insulin resistance and vascularization of thyroid nodules. J Clin Endocrinol Metab.

[30] Marcello MA, Cunha LL, Batista FA, Ward LS (2014). Obesity and thyroid cancer. Endocr Relat Cancer.

[31] Pappa T, Alevizaki M (2014). Obesity and thyroid cancer: a clinical update. Thyroid.

