# Supplementary material for: Association of thyroid nodules with adiposity: a community-based cross-sectional study in China
Source: BMC Endocr Disord. 2018 Jan 27;18:3. doi: 10.1186/s12902-018-0232-8 (PMC5787304; doi:10.1186/s12902-018-0232-8)
Supplement: Supplementary file 1 — Analysis of associations of thyroid nodules with different BMI cut-offs. (DOCX 19 kb) [file 12902_2018_232_MOESM1_ESM.docx]

**Supplementary Table 1:** Analysis of associations of thyroid nodules with different BMI cut-offs.

| **BMI cut-offs** | **Unadjusted OR, 95%CI** | ***P*** | ^†^**Adjusted OR, 95%CI** | ***P*** |
| --- | --- | --- | --- | --- |
| **China Criteria** |  |  |  |  |
| <24 kg/m^2^ | Ref |  | Ref |  |
| ≥24 kg/m^2^ | 1.59 (1.17, 2.16) | 0.003 | 1.33 (0.95, 1.86) | 0.100 |
| **ADA Criteria** |  |  |  |  |
| <23 kg/m^2^ | Ref |  | Ref |  |
| ≥23, <25 kg/m^2^ | 1.62 (1.10, 2.40) | 0.016 | 1.47 (0.97, 2.23) | 0.072 |
| ≥25 kg/m^2^ | 1.70 (1.19, 2.44) | 0.004 | 1.43 (0.96, 2.13) | 0.079 |

^†^The adjusted OR controls for age, gender, education, profession, smoking status, systolic and diastolic blood pressure, TSH, and UIC.
