# Supplementary material for: Association of thyroid nodules with adiposity: a community-based cross-sectional study in China
Source: BMC Endocr Disord. 2018 Jan 27;18:3. doi: 10.1186/s12902-018-0232-8 (PMC5787304; doi:10.1186/s12902-018-0232-8)
Supplement: Supplementary file 2 — Associations of thyroid nodules with different BMI cut-offs in subgroups of subjects stratified by gender, age, TSH or UIC. (DOCX 16 kb) [file 12902_2018_232_MOESM2_ESM.docx]

**Supplementary Table 2:** Associations of thyroid nodules with different BMI cut-offs in subgroups of subjects stratified by gender, age, TSH or UIC.

| **Predictor** | **N** | **China BMI cutoff (kg/m^2^)** | **Adjusted OR, 95%CI** | ***P*** | ***P* interaction** | **ADA BMI cutoff (kg/m^2^)** | **Adjusted OR, 95%CI** | ***P*** | ***P* interaction** |
| --- | --- | --- | --- | --- | --- | --- | --- | --- | --- |
| **Gender^†^** |  |  |  |  |  |  |  |  |  |
| Male | 682 | ≥24 | 1.14 (0.68, 1.90) | 0.626 | 0.372 | ≥23,<25 | 1.71 (0.89, 3.31) | 0.110 | 0.245 |
|  |  |  |  |  |  | ≥25 | 1.10 (0.60, 2.04) | 0.751 |  |
| Female | 800 | ≥24 | **1.61 (1.01, 2.54)** | **0.044** |  | ≥23,<25 | 1.43 (0.81, 2.53) | 0.211 |  |
|  |  |  |  |  |  | ≥25 | **1.95 (1.14, 3.34)** | **0.015** |  |
| **Age^††^** |  |  |  |  |  |  |  |  |  |
| <50 years | 958 | ≥24 | 1.61 (0.94, 2.74) | 0.081 | 0.185 | ≥23,<25 | 1.66 (0.89, 3.09) | 0.110 | 0.749 |
|  |  |  |  |  |  | ≥25 | 1.59 (0.84, 3.00) | 0.152 |  |
| ≥50 years | 524 | ≥24 | 1.14 (0.74, 1.75) | 0.559 |  | ≥23,<25 | 1.37 (0.79, 2.40) | 0.266 |  |
|  |  |  |  |  |  | ≥25 | 1.19 (0.72, 1.96) | 0.502 |  |
| **TSH^†††^** |  |  |  |  |  |  |  |  |  |
| ≤4.2 mIU/L | 1259 | ≥24 | 1.17 (0.81, 1.68) | 0.408 | 0.161 | ≥23,<25 | 1.19 (0.76, 1.88) | 0.440 | **0.044** |
|  |  |  |  |  |  | ≥25 | 1.12 (0.73, 1.71) | 0.594 |  |
| >4.2 mIU/L | 223 | ≥24 | 2.52 (0.91, 6.99) | 0.076 |  | ≥23,<25 | **5.59 (1.39, 22.51)** | **0.015** |  |
|  |  |  |  |  |  | ≥25 | **5.15 (1.30, 20.37)** | **0.020** |  |
| **UIC^††††^** |  |  |  |  |  |  |  |  |  |
| <200 𝜇g/L | 543 | ≥24 | 1.08 (0.63, 1.85) | 0.785 | 0.375 | ≥23,<25 | 1.24 (0.64, 2.41) | 0.529 | 0.833 |
|  |  |  |  |  |  | ≥25 | 1.25 (0.66, 2.35) | 0.494 |  |
| ≥200 𝜇g/L | 939 | ≥24 | 1.48 (0.95, 2.29) | 0.082 |  | ≥23,<25 | 1.61 (0.93, 2.77) | 0.088 |  |
|  |  |  |  |  |  | ≥25 | 1.47 (0.88, 2.46) | 0.144 |  |

^†^ Adjusted for age, education, profession, smoking status, systolic and diastolic blood pressure, TSH, and UIC;

^††^ adjusted for gender, education, profession, smoking status, systolic and diastolic blood pressure, TSH, and UIC;

^†††^ adjusted for age, gender, education, profession, smoking status, systolic and diastolic blood pressure, and UIC;

^††††^ adjusted for age, gender, education, profession, smoking status, systolic and diastolic blood pressure, and TSH.
